# Mollusca collected by Agassiz trawl from the 2016 SO-AntEco (JR15005) expedition to the South Orkney Islands, Antarctica - data

**DOI:** 10.3897/BDJ.11.e105888

**Published:** 2023-10-17

**Authors:** Jan Steger, Katrin Linse, Yi-Ming Gan, Huw J. Griffiths

**Affiliations:** 1 Department of Palaeontology, University of Vienna, Vienna, Austria Department of Palaeontology, University of Vienna Vienna Austria; 2 British Antarctic Survey, Cambridge, United Kingdom British Antarctic Survey Cambridge United Kingdom; 3 Royal Belgian Institute of Natural Sciences, Brussels, Belgium Royal Belgian Institute of Natural Sciences Brussels Belgium

**Keywords:** benthic biodiversity, Southern Ocean, continental slope, continental shelf, Bivalvia, Gastropoda, Polyplacophora, Scaphopoda, Solenogastres

## Abstract

**Background:**

This dataset contributes to the knowledge of macro- and megafaunal Mollusca associated with a range of benthic habitat types in the South Orkney Islands, Antarctica, an exceptionally diverse region of the Southern Ocean. The information presented is derived from Agassiz trawl samples collected on the archipelago’s shelf plateau and slope, within and outside of the South Orkney Islands Southern Shelf Marine Protected Area (SOISS MPA). Sampling was conducted in the framework of the British Antarctic Survey/SCAR “South Orkneys - State of the Antarctic Ecosystem” (SO-AntEco) project aboard RRS *James Clark Ross* during expedition JR15005 in Austral summer 2016. This dataset is published by the British Antarctic Survey under the licence CC-BY 4.0. We would appreciate it if you could follow the guidelines from the SCAR Data Policy (SCAR 2023) when using the data. If you have any questions regarding this dataset, do not hesitate to contact us via the contact information provided in the metadata or via data-biodiversity-aq@naturalsciences.be. Issues with the dataset can be reported at https://github.com/biodiversity-aq/data-publication/. This dataset is part of the Biodiversity, Evolution and Adaptation Project of the Environmental Change and Evolution Program of the British Antarctic Survey. The cruise report of the expedition is available at https://www.bodc.ac.uk/resources/inventories/cruise_inventory/reports/jr15005.pdf.

**New information:**

We report occurrences of Mollusca from individual samples taken with a 2 m-wide Agassiz trawl (AGT) in the framework of the February – March 2016 research expedition JR15005 of RRS *James Clark Ross* to the SOISS MPA and adjacent shelf and slope areas. Of 78 successful AGT deployments, 44 trawls at depths ranging from 235-2194 m yielded living Mollusca, totalling 2276 individuals, 67 morphospecies and 163 distributional records. One hundred and fifteen empty shells were also collected and recorded in the dataset. Three morphospecies (one Bivalvia and two Gastropoda) were sampled exclusively as empty shells, yielding a total of 70 morphospecies and 2391 specimens represented in the dataset. All specimens were preserved in 96% undenatured ethanol and are stored as vouchers in the collections of the British Antarctic Survey (BAS), Cambridge, United Kingdom. The publication of this dataset aims at increasing the knowledge on the biodiversity, abundance and geographical and bathymetric distribution of larger-sized epi- and shallow infaunal Mollusca of the South Orkney Islands.

## Introduction

The aim of the SO-AntEco expedition (JR15005) was to assess and contribute to a better understanding of the benthic biodiversity, community composition and zonation on the shelf and slope areas of the South Orkney Islands, including the SOISS MPA, and to provide information for future MPA monitoring and management. Previous studies have shown the archipelago to host an exceptionally high biodiversity for a polar site, most of which is contributed by marine benthic species (Barnes et al. 2009). To protect representative examples of marine ecosystems, habitats, and the biodiversity in the region, CCAMLR closed all demersal finfish fisheries around the islands in 1989 and established the SOISS MPA in 2009 (CCAMLR-XXVIII paragraph 7.1; CM 91-03; CCAMLR (2009)), the world's first MPA located entirely within the high seas (Brasier et al. 2018). Benthic sampling stations in the present dataset ranged from approximately 200 to 2000 m water depth and were chosen to cover different habitats, informed by the map of geomorphic zones by Dickens et al. (2014). These habitats included: (i) shelf, (ii) gentle shelf slope, and (iii) ridge along the southern shelf edge of the South Orkney Islands; and (i) shelf, (ii) steep shelf slope, (iii) marginal plateau, (iv) seamount, and (v) steep trough walls along the northern shelf edge (Brasier et al. 2018).

We here report on the molluscs collected during the JR15005 campaign as they are a taxonomically and ecologically diverse phylum known to be a good surrogate taxon for entire benthic communities (Smith 2005, Tyler and Kowalewski 2017). With almost 700 formally described species in the Southern Ocean (Clarke and Johnston 2003), Mollusca is one of the more speciose higher-level taxa in the region. However, even though the group has been the target of investigations since the early Antarctic expeditions and, thus, is amongst the best known in the Southern Ocean (Clarke and Johnston 2003, De Broyer and Danis 2011), recent investigations in yet un(der)studied habitats, regions and depths have continued to record or describe new species on a regular basis (e.g., Aldea et al. (2009), Zamarro et al. (2013), Chen et al. (2015), Kantor et al. (2016)).

In this context, the publication of this dataset aims at increasing the knowledge on the macro- and megabenthic molluscs from the study area that encompasses the shelf edge and little investigated slope areas of the South Orkney Islands archipelago, Scotia Arc, in the Atlantic sector of the Southern Ocean.

## Project description

### Title

Mollusca collected by Agassiz trawl from the 2016 SO-AntEco expedition to the South Orkney Islands, Antarctica – data

### Personnel

Jan Steger, Katrin Linse, Yi-Ming Gan, Huw J. Griffiths

### Funding

The SO-AntEco - JR15005 expedition was part of the Biodiversity, Evolution and Adaptation Project of the Environmental Change and Evolution Program of the British Antarctic Survey (BAS). The publication of this dataset was funded by the Belgian Science Policy Office (BELSPO, contract n°FR/36/AN1/AntaBIS) in the framework of EU-LifeWatch as a contribution to the SCAR Antarctic Biodiversity Portal (biodiversity.aq).

## Sampling methods

### Study extent

**Figure 1.**
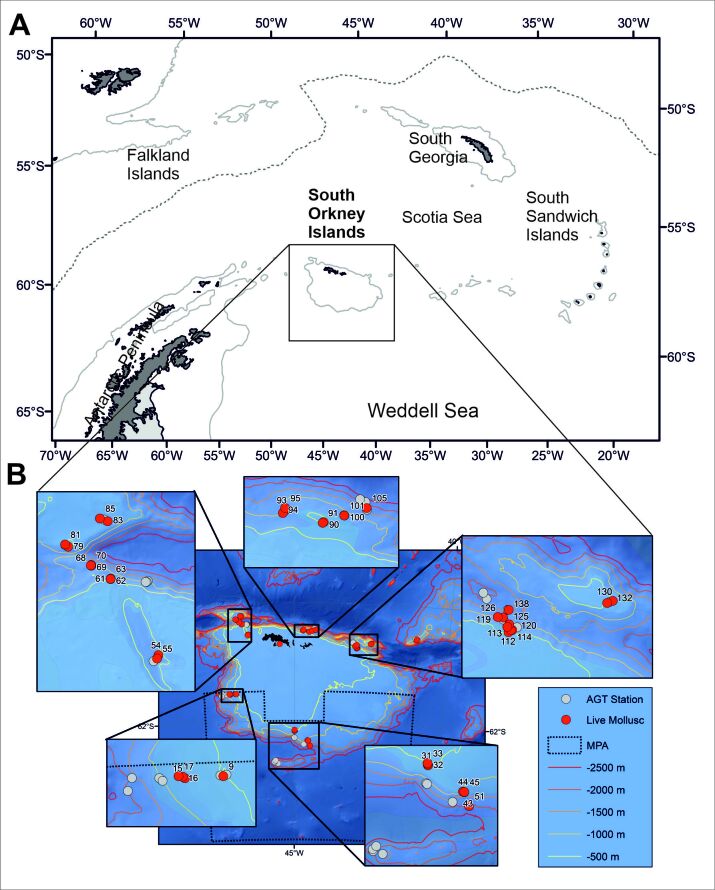
Map of the South Orkney Islands and sampling stations. **A** Location of the South Orkney Islands in the Scotia Sea, Southern Ocean. **B** Positions of Agassiz trawl (AGT) sampling stations of the SO-AntEco project along the shelf and slope areas of the archipelago. The boundaries of the South Orkney Islands Southern Shelf Marine Protected Area (SOISS MPA) are indicated by a dotted line. Sites that yielded living Mollusca are highlighted as red dots, grey dots mark the positions of trawls that did not contain any molluscs. Findings of empty shells were limited to trawl stations where also living molluscs were found.

The study area encompasses the continental shelf and slope of the South Orkney Islands, Scotia Arc, in the Atlantic sector of the Southern Ocean (Fig. 1A). The archipelago is situated approximately 600 km north-east of the tip of the Antarctic Peninsula and hydrographically affected by the Antarctic Circumpolar Current in the north and the Weddell Sea Gyre in the south (Barnes et al. 2009, Meredith et al. 2011, Dickens et al. 2014). The present dataset provides species occurrences and specimen counts of larger-sized (mostly > 1 cm) Mollusca from 44 AGT hauls (= stations) taken during RRS *James Clark Ross* cruise JR15005 in 2016. Detailed station information is provided in Table 1, their geographic positions are plotted in Fig. 1B.

### Sampling description

Mollusca were collected using a 2 m-wide AGT with an outer 3 cm mesh-sized and an inner 1 cm mesh-sized net following a standardised deployment protocol (see JR15005 cruise report at https://www.bodc.ac.uk/resources/inventories/cruise_inventory/report/16041/).

Six major clusters of sampling stations were located to the south, west, northwest (subdivided into two sites: “North West” and “North West Trough”), north and northeast of the South Orkney Islands, as well as at Bruce Bank. Targeted sampling depths were 500, 750, 1000, 1500 and 2000 m. Furthermore, a single trawl was taken on the shelf off Signy Island at approximately 200 m depth. If conditions permitted, three replicate AGTs were taken at the shallower stations (500, 750 and 1000 m), while at least one haul was conducted at the deeper stations (1500 and 2000 m). Detailed locations of major clusters and individual sampling sites are shown in Fig. 1B. Swath multibeam bathymetry was used to assess seafloor topography prior to AGT deployments and – if possible (at < 1000 m water depth) – *in situ* seafloor imaging conducted at the same sites using the shallow underwater camera system (SUCS) to gather additional information on local faunal density, biomass and abundance (see also Brasier et al. (2018)).

Once on the bottom, the AGT was towed for 10 min at a speed of 1 kn. On deck, samples were sorted by hand into higher-level taxonomic groups (e.g., molluscan classes), the collected individuals roughly counted, placed in pre-cooled 96% ethanol and stored at -20°C. All molluscan specimens recovered during JR15005 were preserved.

**Treatment of samples**: Identification of specimens was performed at BAS, based on external morphological characters using specialised literature (e.g., Dell (1990), Hain (1990), Numanami (1996), Steiner and Linse (2000), Aldea and Troncoso (2008), Engl (2012), Kantor et al. (2016)) and an available reference collection. Mollusca were identified to the lowest possible taxonomic level, excluding one live collected individual and shell fragments too damaged for identification. Thirty-four percent of morphospecies (24 out of 70 species, Table 2) could only be classified at genus level or above: two of these were Solenogastres, two Polyplacophora, one Bivalvia and 19 Gastropoda. Taxa not identified to species level represent distinct morphospecies and do not belong to any other species in the dataset. Further morphological and genetic studies are required to clarify the status of these entities. Of morphospecies likely to represent undescribed species, DNA has been extracted for future COI sequence analysis. Three of the 70 morphospecies (*Cuspidariatenella* E. A. Smith, 1907, *Torellia* sp. 1 [the sole representative of family Capulidae in our material] and Falsilunatiacf.scotiana) were collected only as empty shells.

Each dataset entry corresponds to either live collected individuals or empty shells of a single molluscan species from a particular station stored within a labelled container/vial in the BAS voucher collection. Multiple conspecific specimens from any one sampling event may be distributed across more than one container (see Table 3 for a detailed breakdown).

### Quality control

All records were validated. Coordinates were plotted on a map to verify that the actual geographical location corresponds to its locality. Event dates and time were converted into ISO 8601 and verified with the field reports. All scientific names were checked for typos and matched to the species information backbone of the World Register of Marine Species (WoRMS Editorial Board (2018); http://marinespecies.org/); Life Science Identifiers (LSIDs) were assigned to each taxon as scientificNameID.

### Step description

1. Benthic sampling with a 2 m-wide AGT in the South Orkney Islands during research cruise JR15005.
2. On board, the catch was hand-sorted into different higher-level taxa, with Mollusca segregated mostly to class level, and animals subsequently fixed in 96% ethanol at -20°C.
3. Taxonomic identification of the molluscan specimens was performed at the British Antarctic Survey (Cambridge, United Kingdom), based on morphological characters and with the aid of a stereomicroscope.
4. Species-level specimen counts were compiled for each trawl sample (Table 3).
5. Of species potentially new to science, DNA was extracted for future molecular analysis.

## Geographic coverage

### Description

Continental shelf and slope of the South Orkney Islands, Southern Ocean.

### Coordinates

-62.4202 and -60.2135 Latitude; -47.1704 and -41.0376 Longitude.

## Taxonomic coverage

### Description

The present dataset provides information on the phylum Mollusca and includes five classes (Aplacophora, Polyplacophora, Bivalvia, Gastropoda and Scaphopoda). In total, 2276 living individuals were collected, representing 36 families, 67 species and 163 species-level distributional records (Table 3). To date, two morphospecies of the aplacophoran taxon Solenogastres, one morphospecies of an unidentified conoidean neogastropod and three morphospecies of nudibranchs (Gastropoda) have not been assigned to a family. Of live collected individuals, 1999 specimens (88%) were Bivalvia (accounting for 15 species), 158 (7%) Gastropoda (accounting for 45 species), 113 (5%) Scaphopoda (accounting for two species), four (0.2%) Polyplacophora (accounting for three species) and two (0.1%) Aplacophora (accounting for two species). Furthermore, 115 empty shells are recorded in the dataset. One family (Capulidae) and three species-level taxa (the cuspidariid bivalve *Cuspidariatenella* E. A. Smith, 1907, the capulid gastropod *Torellia* sp. 1 and the naticid gastropod Falsilunatiacf.scotiana) were exclusively recorded as empty shells (Table 3).

## Temporal coverage

**Data range:** 2016-2-29 – 2016-3-19.

## Usage licence

### Usage licence

Other

### IP rights notes

This work is licensed under a Creative Commons Attribution (CC-BY) 4.0 Licence.

## Data resources

### Data package title

Mollusca collected by Agassiz trawl from the 2016 SO-AntEco expedition to the South Orkney Islands, Antarctica - data

### Resource link

https://doi.org/10.15468/hza883

### Alternative identifiers

https://ipt.biodiversity.aq/resource?r=bas_jr15005_molluscs

### Number of data sets

1

### Data set 1.

#### Data set name

Mollusca collected by Agassiz trawl from the 2016 SO-AntEco expedition to the South Orkney Islands, Antarctica - data

#### Data format

Darwin Core

#### Description

The dataset, published by Steger et al. (2023), contains information on specimens of Southern Ocean Mollusca that were collected by Agassiz trawl (AGT) during the 2016 JR15005 SO-AntEco research cruise. The specific aims and objectives of the cruise can be found in the cruise report (available at https://www.bodc.ac.uk/resources/inventories/cruise_inventory/report/16041/). The dataset and associated specimens will be used to investigate the biodiversity and assemblage structure of molluscs on the South Orkney Islands shelf and slope and to identify new species records from yet un(der)sampled depths, as well as undescribed species. This dataset is published by the British Antarctic Survey under the licence CC-BY 4.0. The publication of this dataset was funded by the Belgian Science Policy Office (BELSPO, contract n°FR/36/AN1/AntaBIS) in the framework of EU-LifeWatch as a contribution to the SCAR Antarctic Biodiversity Portal (biodiversity.aq). Please follow the guidelines from the SCAR Data Policy (SCAR 2023) when using the data. If you have any questions regarding this dataset, please do not hesitate to contact us via the contact information provided in the metadata or via data-biodiversity-aq@naturalsciences.be.

The dataset column descriptions below are modified after Wieczorek et al. (2012) and Darwin Core Maintenance Group (2021).

**Data set 1.** 

Column label	Column description
id	Identifier of a record, consisting of the cruise number (JR15005), underscore, 4-digit vial number as assigned in the field, dot, alphanumeric morphospecies code, underscore and a single letter indicating whether the record pertains to live collected individuals (“A”) or empty shells (“D”).
type	The nature or genre of the resource.
language	Language of the resource.
institutionID	An identifier for the institution having custody of the object(s) referred to in the record.
institutionCode	The acronym in use by the institution having custody of the object(s) referred to in the record.
basisOfRecord	The specific nature of the data record.
occurrenceID	An identifier for the Occurrence (as opposed to a particular digital record of the Occurrence). For details, see description of column id.
catalogNumber	An identifier for the record within the dataset or collection. For details, see description of column id.
organismQuantity	Number of specimens. Loose valves of bivalves were counted as one specimen each.
organismQuantityType	The type of quantification system used for the quantity of Organisms.
occurrenceStatus	A statement about the presence or absence of a Taxon at a Location.
preparations	A list (concatenated and separated) of preparations and preservation methods for a specimen.
occurrenceRemarks	Comments or notes about the Occurrence. Indicates whether specimen(s) was/were live collected or dead (i.e., empty shells).
eventID	An identifier for the set of information associated with an Event (something that occurs at a place and time) – RRS *James Clark Ross* JR15005 station number.
fieldNumber	An identifier given to the Event in the field.
eventDate	The date-time when the Event was recorded.
year	The four-digit year in which the Event occurred, according to the Common Era Calendar.
month	The integer month in which the Event occurred.
day	The integer day of the month on which the Event occurred.
verbatimEventDate	The verbatim original representation of the date and time information for an Event.
samplingProtocol	The names of, references to, or descriptions of the methods or protocols used during an Event.
countryCode	The standard code for the country in which the Location occurs.
locality	The specific description of the place.
minimumDepthInMeters	The lesser depth of a range of depth for the Event below the local surface, in metres.
maximumDepthInMeters	The greater depth of a range of depth for the Event below the local surface, in metres.
decimalLatitude	The geographic latitude (in decimal degrees, using the spatial reference system given in geodeticDatum) of the geographic centre of a Location. Positive values are north of the equator, negative values are south of it.
decimalLongitude	The geographic longitude (in decimal degrees, using the spatial reference system given in geodeticDatum) of the geographic centre of a Location. Positive values are east of the Greenwich Meridian, negative values are west of it.
geodeticDatum	The ellipsoid, geodetic datum, or spatial reference system (SRS) upon which the geographic coordinates given in decimalLatitude and decimalLongitude are based.
coordinateUncertaintyInMeters	The horizontal distance (in metres) from the given decimalLatitude and decimalLongitude describing the smallest circle containing the whole of the Location.
coordinatePrecision	A decimal representation of the precision of the coordinates given in the decimalLatitude and decimalLongitude.
footprintWKT	A Well-Known Text (WKT) representation of the shape (footprint, geometry) that defines the Location.
footprintSRS	The ellipsoid, geodetic datum, or spatial reference system (SRS) upon which the geometry given in footprintWKT is based.
verbatimIdentification	A string representing the taxonomic identification as it appeared in the original record.
identificationQualifier	A brief phrase or a standard term ("cf.", "aff.") to express the determiner's doubts about the Identification.
identifiedBy	A list (concatenated and separated) of names of people, groups, or organisations who assigned the Taxon to the subject.
identifiedByID	A list (concatenated and separated) of the globally unique identifier (ORCID iD) for the person, people, groups, or organisations responsible for assigning the Taxon to the subject.
identificationRemarks	Comments or notes about the Identification.
scientificNameID	Taxon number (Life Science Identifier, LSID) according to the World Register of Marine Species (https://www.marinespecies.org).
scientificName	The full scientific name, corresponding to the lowest level taxonomic rank that can be determined.
kingdom	The full scientific name of the kingdom in which the Taxon is classified.
phylum	The full scientific name of the phylum in which the Taxon is classified.
class	The full scientific name of the class in which the Taxon is classified.
order	The full scientific name of the order in which the Taxon is classified.
family	The full scientific name of the family in which the Taxon is classified.
genus	The full scientific name of the genus in which the Taxon is classified.
specificEpithet	The name of the first or species epithet of the scientificName.
taxonRank	The taxonomic rank of the most specific name in the scientificName.
scientificNameAuthorship	The authorship information for the scientificName formatted according to the conventions of the applicable nomenclaturalCode.

## Figures and Tables

**Table 1.** Details on SO-AntEco/JR15005 Agassiz trawl stations that yielded Mollusca. Positions are given according to datum WGS84, depths in metres, dates in the format dd.mm.yyyy.

**Station No.**	**Start Lat.**	**End Lat.**	**Start Long.**	**End Long.**	**Min. Depth**	**Max. Depth**	**Region**	**Date**
JR15005_6	-60.6997	-60.6988	-45.4727	-45.4756	235	243	Signy	29.02.2016
JR15005_9	-61.5357	-61.5339	-46.9372	-46.9458	522	564	West	02.03.2016
JR15005_15	-61.5374	-61.5343	-47.1298	-47.1375	772	791	West	03.03.2016
JR15005_16	-61.5332	-61.5311	-47.1406	-47.1538	791	817	West	03.03.2016
JR15005_17	-61.5318	-61.5300	-47.1614	-47.1704	836	844	West	03.03.2016
JR15005_31	-62.1681	-62.1643	-44.9830	-44.9861	531	535	South	05.03.2016
JR15005_32	-62.1617	-62.1569	-44.9877	-44.9883	524	530	South	05.03.2016
JR15005_33	-62.1542	-62.1498	-44.9902	-44.9877	517	556	South	06.03.2016
JR15005_43	-62.3337	-62.3316	-44.5258	-44.5351	1000	1000	South	06.03.2016
JR15005_44	-62.3302	-62.3278	-44.5417	-44.5532	969	1012	South	06.03.2016
JR15005_45	-62.3329	-62.3287	-44.5262	-44.5345	1006	1055	South	07.03.2016
JR15005_51	-62.4202	-62.4185	-44.4629	-44.4801	2098	2194	South	08.03.2016
JR15005_54	-60.5341	-60.5386	-46.4837	-46.4879	785	807	NW Trough	09.03.2016
JR15005_55	-60.5419	-60.5465	-46.4903	-46.4939	808	810	NW Trough	09.03.2016
JR15005_61	-60.3547	-60.3588	-46.6842	-46.6885	465	540	North West	09.03.2016
JR15005_62	-60.3539	-60.3583	-46.6841	-46.6875	458	573	North West	10.03.2016
JR15005_63	-60.3544	-60.3589	-46.6842	-46.6876	442	545	North West	10.03.2016
JR15005_68	-60.3224	-60.3262	-46.7672	-46.7726	732	852	North West	10.03.2016
JR15005_69	-60.3208	-60.3254	-46.7674	-46.7704	733	837	North West	10.03.2016
JR15005_70	-60.3216	-60.3266	-46.7690	-46.7699	722	826	North West	10.03.2016
JR15005_79	-60.2766	-60.2742	-46.8659	-46.8739	484	486	North West	11.03.2016
JR15005_81	-60.2713	-60.2695	-46.8784	-46.8870	459	485	North West	11.03.2016
JR15005_83	-60.2207	-60.2182	-46.6898	-46.6988	783	807	North West	11.03.2016
JR15005_85	-60.2135	-60.2158	-46.7234	-46.7332	798	838	North West	12.03.2016
JR15005_90	-60.4944	-60.4965	-44.5179	-44.5255	520	624	North	12.03.2016
JR15005_91	-60.4959	-60.4988	-44.5224	-44.5297	551	658	North	13.03.2016
JR15005_93	-60.4641	-60.4687	-44.7027	-44.7112	1028	1077	North	13.03.2016
JR15005_94	-60.4717	-60.4753	-44.7158	-44.7260	992	1028	North	13.03.2016
JR15005_95	-60.4589	-60.4562	-44.7044	-44.7146	1062	1149	North	13.03.2016
JR15005_100	-60.4763	-60.4719	-44.4192	-44.4187	790	896	North	13.03.2016
JR15005_101	-60.4768	-60.4726	-44.4190	-44.4224	773	858	North	13.03.2016
JR15005_105	-60.4572	-60.4550	-44.3115	-44.3257	1447	1483	North	14.03.2016
JR15005_112	-60.7522	-60.7565	-42.9576	-42.9693	548	598	North East	15.03.2016
JR15005_113	-60.7578	-60.7595	-42.9744	-42.9844	508	546	North East	15.03.2016
JR15005_114	-60.7524	-60.7531	-42.9632	-42.9734	1758	2190	North East	15.03.2016
JR15005_119	-60.7387	-60.7348	-42.9725	-42.9802	801	867	North East	15.03.2016
JR15005_120	-60.7450	-60.7405	-42.9811	-42.9881	708	831	North East	15.03.2016
JR15005_125	-60.7222	-60.7190	-43.0022	-43.0125	1034	1139	North East	16.03.2016
JR15005_126	-60.7216	-60.7205	-43.0267	-43.0365	1014	1017	North East	16.03.2016
JR15005_130	-60.6713	-60.6728	-42.4951	-42.5034	512	531	North East	16.03.2016
JR15005_132	-60.6772	-60.6786	-42.5206	-42.5295	493	500	North East	16.03.2016
JR15005_138	-60.7032	-60.6978	-42.9774	-42.9901	1541	1674	North East	17.03.2016
JR15005_142	-60.5733	-60.5715	-41.0549	-41.0633	621	651	Bruce Bank	18.03.2016
JR15005_146	-60.5589	-60.5617	-41.0376	-41.0461	622	625	Bruce Bank	19.03.2016

**Table 2.** Distribution of the 2391 molluscan specimens in the dataset across families, genera, and species. Specimen counts (# spcms.) presented subsume live and dead collected specimens. For a detailed breakdown of specimen counts by station and into live individuals and empty shells, see Table 3.

**Family**	**Genus**	**Species**	# **spcms.**
		Solenogastres sp. 1	1
		Solenogastres sp. 2	1
Leptochitonidae			3
Leptochitonidae	*Leptochiton*		3
Leptochitonidae	*Leptochiton*	*Leptochiton* sp. 1	2
Leptochitonidae	*Leptochiton*	*Leptochiton* sp. 2	1
Mopaliidae			1
Mopaliidae	*Nuttallochiton*		1
Mopaliidae	*Nuttallochiton*	*Nuttallochitonmirandus*	1
Nuculanidae			76
Nuculanidae	*Nuculana*		56
Nuculanidae	*Nuculana*	*Nuculanainaequisculpta*	56
Nuculanidae	*Propeleda*		20
Nuculanidae	*Propeleda*	*Propeledalongicaudata*	20
Yoldiidae			7
Yoldiidae	*Yoldiella*		7
Yoldiidae	*Yoldiella*	Yoldiellacf.valettei	7
Sareptidae			17
Sareptidae	*Aequiyoldia*		17
Sareptidae	*Aequiyoldia*	*Aequiyoldiaeightsii*	17
Limopsidae			49
Limopsidae	*Limopsis*		49
Limopsidae	*Limopsis*	*Limopsismarionensis*	49
Philobryidae			1856
Philobryidae	*Adacnarca*		1
Philobryidae	*Adacnarca*	*Adacnarcanitens*	1
Philobryidae	*Lissarca*		1855
Philobryidae	*Lissarca*	*Lissarcanotorcadensis*	1855
Lasaeidae			3
Lasaeidae	*Kellia*		1
Lasaeidae	*Kellia*	*Kellia* sp. 1	1
Lasaeidae	*Altenaeum*		2
Lasaeidae	*Altenaeum*	*Altenaeumgibbosum*	2
Thyasiridae			21
Thyasiridae	*Thyasira*		18
Thyasiridae	*Thyasira*	*Thyasiradebilis*	3
Thyasiridae	*Thyasira*	*Thyasirafalklandica*	15
Thyasiridae	*Parathyasira*		3
Thyasiridae	*Parathyasira*	*Parathyasiradearborni*	3
Thraciidae			1
Thraciidae	*Thracia*		1
Thraciidae	*Thracia*	*Thraciameridionalis*	1
Cuspidariidae			7
Cuspidariidae	*Cuspidaria*		7
Cuspidariidae	*Cuspidaria*	*Cuspidariainfelix*	4
Cuspidariidae	*Cuspidaria*	*Cuspidariaminima*	1
Cuspidariidae	*Cuspidaria*	*Cuspidariatenella*	2
Eucyclidae			2
Eucyclidae	*Calliotropis*		2
Eucyclidae	*Calliotropis*	*Calliotropiseltanini*	1
Eucyclidae	*Calliotropis*	*Calliotropislateumbilicata*	1
Calliostomatidae			1
Calliostomatidae	*Falsimargarita*		1
Calliostomatidae	*Falsimargarita*	*Falsimargaritathielei*	1
Colloniidae			2
Colloniidae	*Leptocollonia*		2
Colloniidae	*Leptocollonia*	*Leptocollonia* sp. 1	2
Capulidae			1
Capulidae	*Torellia*		1
Capulidae	*Torellia*	*Torellia* sp. 1	1
Naticidae			16
Naticidae	*Amauropsis*		2
Naticidae	*Amauropsis*	*Amauropsisaureolutea*	2
Naticidae	*Falsilunatia*		11
Naticidae	*Falsilunatia*	Falsilunatiacf.scotiana	1
Naticidae	*Falsilunatia*	*Falsilunatia* sp. 1	10
Naticidae		Naticidae cf. *Falsilunatia* sp. 1	2
Naticidae	*Sinuber*		1
Naticidae	*Sinuber*	*Sinuber* sp. 1	1
Velutinidae			3
Velutinidae	*Marseniopsis*		3
Velutinidae	*Marseniopsis*	*Marseniopsis* sp. 1	1
Velutinidae	*Marseniopsis*	*Marseniopsis* sp. 2	1
Velutinidae	*Marseniopsis*	*Marseniopsis* sp. 3	1
Prosiphonidae			48
Prosiphonidae	*Antarctodomus*		1
Prosiphonidae	*Antarctodomus*	*Antarctodomusthielei*	1
Prosiphonidae	*Chlanidota*		27
Prosiphonidae	*Chlanidota*	*Chlanidotasigneyana*	27
Prosiphonidae	*Chlanificula*		15
Prosiphonidae	*Chlanificula*	*Chlanificulathielei*	15
Prosiphonidae	*Neobuccinum*		4
Prosiphonidae	*Neobuccinum*	*Neobuccinumeatoni*	4
Prosiphonidae	*Probuccinum*		1
Prosiphonidae	*Probuccinum*	*Probuccinumtenerum*	1
Cominellidae			2
Cominellidae	*Falsitromina*		2
Cominellidae	*Falsitromina*	*Falsitromina* sp. 1	2
Muricidae			5
Muricidae	*Trophon*		5
Muricidae	*Trophon*	*Trophoncoulmanensis*	4
Muricidae	*Trophon*	*Trophon* sp. 1	1
Marginellidae			4
Marginellidae	*Volvarina*		4
Marginellidae	*Volvarina*	*Volvarinaealesae*	3
Marginellidae	*Volvarina*	*Volvarinahyalina*	1
Volutidae			38
Volutidae	*Harpovoluta*		33
Volutidae	*Harpovoluta*	*Harpovolutacharcoti*	33
Volutidae	*Miomelon*		4
Volutidae	*Miomelon*	*Miomelonturnerae*	4
Volutidae	*Tractolira*		1
Volutidae	*Tractolira*	*Tractolira* sp. 1	1
Volutomitridae			17
Volutomitridae	*Paradmete*		17
Volutomitridae	*Paradmete*	Paradmetecf.fragillima	6
Volutomitridae	*Paradmete*	*Paradmetepercarinata*	10
Volutomitridae	*Paradmete*	*Paradmete* sp. 1	1
Borsoniidae			14
Borsoniidae	*Belaturricula*		4
Borsoniidae	*Belaturricula*	Belaturriculacf.ergata	1
Borsoniidae	*Belaturricula*	*Belaturriculagaini*	3
Borsoniidae	*Typhlodaphne*		10
Borsoniidae	*Typhlodaphne*	*Typhlodaphneparatenoceras*	10
		Neogastropoda cf. Borsoniidae sp. 1	2
Mangeliidae			1
Mangeliidae	*Lorabela*		1
Mangeliidae	*Lorabela*	*Lorabelapelseneeri*	1
Raphitomidae			1
Raphitomidae	*Pleurotomella*		1
Raphitomidae	*Pleurotomella*	Pleurotomellacf.simillima	1
Cochlespiridae			15
Cochlespiridae	*Aforia*		15
Cochlespiridae	*Aforia*	*Aforiamagnifica*	15
Pseudomelatomidae			23
Pseudomelatomidae	*Conorbela*		23
Pseudomelatomidae	*Conorbela*	*Conorbelaantarctica*	23
Horaiclavidae			9
Horaiclavidae	*Micropleurotoma*		9
Horaiclavidae	*Micropleurotoma*	*Micropleurotoma* sp. 1	9
Cancellariidae			3
Cancellariidae	*Nothoadmete*		3
Cancellariidae	*Nothoadmete*	*Nothoadmeteconsobrina*	3
Acteonidae			3
Acteonidae	*Neactaeonina*		3
Acteonidae	*Neactaeonina*	*Neactaeoninaedentula*	3
Newnesiidae			3
Newnesiidae	*Newnesia*		3
Newnesiidae	*Newnesia*	*Newnesia* sp. 1	3
Philinidae			4
Philinidae	*Philine*		4
Philinidae	*Philine*	*Philine* sp. 1	4
Bathydorididae			2
Bathydorididae	*Prodoris*		2
Bathydorididae	*Prodoris*	Prodoriscf.clavigera	2
Dorididae			5
Dorididae	*Doris*		5
Dorididae	*Doris*	*Doriskerguelenensis*	5
		Nudibranchia sp. 1	2
		Nudibranchia sp. 2	1
		Nudibranchia sp. 3	1
Dentaliidae			15
Dentaliidae	*Dentalium*		15
Dentaliidae	*Dentalium*	*Dentaliummajorinum*	15
Gadilidae			105
Gadilidae	*Siphonodentalium*		105
Gadilidae	*Siphonodentalium*	*Siphonodentaliumdalli*	105

**Table 3.** Species occurrences and specimen counts (# spcms.) for the 44 Agassiz trawl samples (column "Station No.") of the SO-AntEco/JR15005 expedition that yielded Mollusca. For each species, a complete inventory of vials stored in the voucher collection of the British Antarctic Survey (Cambridge, United Kingdom) is provided, including their content in terms of: (i) total number of specimens, (ii) live collected individuals, and (iii) empty shells. In bivalves, a dead specimen may represent either a complete (i.e., articulated) empty shell or a loose valve.

**Species**	# **spcms. (Species)**	**Station No.**	# **spcms. (Station)**	**Vial ID**	# **spcms. (Vial)**		
					**Total**	**Alive**	**Dead**
Solenogastres sp. 1	1	JR15005_79	1	1615.Sol01	1	1	0
Solenogastres sp. 2	1	JR15005_142	1	2957.Sol02	1	1	0
*Leptochiton* sp. 1	2	JR15005_68	1	1334.P02	1	1	0
		JR15005_70	1	1466.P02	1	1	0
*Leptochiton* sp. 2	1	JR15005_105	1	2218.P03	1	1	0
*Nuttallochitonmirandus*	1	JR15005_81	1	1696.P01	1	1	0
*Nuculanainaequisculpta*	56	JR15005_6	53	0152.B03	52	43	9
				0154.B03	1	0	1
		JR15005_54	2	1032.B03	1	0	1
				1043.B03	1	0	1
		JR15005_63	1	1271.B03	1	1	0
*Propeledalongicaudata*	20	JR15005_61	6	1120.B10	6	4	2
		JR15005_62	1	1195.B10	1	1	0
		JR15005_63	11	1269.B10	11	10	1
		JR15005_70	2	1507.B10	2	0	2
Yoldiellacf.valettei	7	JR15005_6	6	0147.B04	1	1	0
				0154.B04	4	2	2
				0162.B04	1	0	1
		JR15005_54	1	1032.B04	1	0	1
*Aequiyoldiaeightsii*	17	JR15005_6	17	0147.B09	16	10	6
				0162.B09	1	0	1
*Limopsismarionensis*	49	JR15005_62	1	1195.B02	1	1	0
		JR15005_70	3	1459.B02	1	1	0
				1460.B02	1	1	0
				1515.B02	1	1	0
		JR15005_79	1	1600.B02	1	0	1
		JR15005_90	1	1831.B02	1	1	0
		JR15005_91	3	1894.B02	3	3	0
		JR15005_94	21	2906.B02	2	2	0
				2907.B02	19	17	2
		JR15005_119	3	2530.B02	3	3	0
		JR15005_126	1	2730.B02	1	1	0
		JR15005_130	2	2782.B02	2	1	1
		JR15005_132	7	2819.B02	1	1	0
				2840.B02	6	5	1
		JR15005_142	5	2948.B02	5	5	0
		JR15005_146	1	3127.B02	1	1	0
*Adacnarcanitens*	1	JR15005_62	1	1194.B16	1	1	0
*Lissarcanotorcadensis*	1855	JR15005_9	448	0185.B01	448	448	0
		JR15005_15	11	0286.B01	11	11	0
		JR15005_17	4	0343.B01	2	2	0
				0351.B01	2	2	0
		JR15005_31	3	0568.B01	3	3	0
		JR15005_32	6	0621.B01	6	6	0
		JR15005_61	493	1094.B01	38	38	0
				1097.B01	51	51	0
				1120.B01	403	403	0
				1133.B01	1	1	0
		JR15005_62	269	1194.B01	268	268	0
				3258.B01	1	1	0
		JR15005_63	268	1271.B01	268	268	0
		JR15005_83	2	1773.B01	2	2	0
		JR15005_90	7	1838.B01	7	7	0
		JR15005_91	146	1878.B01	144	144	0
				1893.B01	1	1	0
				1894.B01	1	1	0
		JR15005_112	22	2304.B01	22	22	0
		JR15005_114	26	2455.B01	26	26	0
		JR15005_119	96	2530.B01	1	1	0
				2531.B01	95	95	0
		JR15005_120	51	2592.B01	51	51	0
		JR15005_132	3	2862.B01	3	3	0
*Kellia* sp. 1	1	JR15005_6	1	0154.B14	1	1	0
*Altenaeumgibbosum*	2	JR15005_6	2	0154.B13	2	2	0
*Thyasiradebilis*	3	JR15005_6	3	0147.B07	1	1	0
				0162.B07	2	2	0
*Thyasirafalklandica*	15	JR15005_6	12	0154.B11	12	12	0
		JR15005_54	3	1028.B11	3	1	2
*Parathyasiradearborni*	3	JR15005_6	3	0154.B08	2	2	0
				0162.B08	1	1	0
*Thraciameridionalis*	1	JR15005_6	1	0162.B06	1	1	0
*Cuspidariainfelix*	4	JR15005_61	2	1120.B15	2	2	0
		JR15005_62	1	1195.B15	1	0	1
		JR15005_91	1	1894.B15	1	1	0
*Cuspidariaminima*	1	JR15005_6	1	0162.B05	1	1	0
*Cuspidariatenella*	2	JR15005_70	1	1461.B17	1	0	1
		JR15005_130	1	2782.B17	1	0	1
*Calliotropiseltanini*	1	JR15005_146	1	3144.G25	1	1	0
*Calliotropislateumbilicata*	1	JR15005_51	1	0997.G16	1	1	0
*Falsimargaritathielei*	1	JR15005_61	1	1133.G35	1	1	0
*Leptocollonia* sp. 1	2	JR15005_62	1	1163.G07	1	1	0
		JR15005_70	1	1486.G07	1	1	0
*Torellia* sp. 1	1	JR15005_79	1	1600.G30	1	0	1
*Amauropsisaureolutea*	2	JR15005_70	1	1486.G40	1	1	0
		JR15005_79	1	1600.G40	1	0	1
Falsilunatiacf.scotiana	1	JR15005_61	1	1133.G36	1	0	1
*Falsilunatia* sp. 1	10	JR15005_61	3	1106.G41	1	0	1
				1133.G41	2	0	2
		JR15005_62	1	1163.G41	1	0	1
		JR15005_63	3	1264.G41	1	0	1
				1270.G41	2	1	1
		JR15005_70	3	1472.G41	1	1	0
				1486.G41	2	1	1
Naticidae cf. *Falsilunatia* sp. 1	2	JR15005_61	1	1133.G42	1	1	0
		JR15005_63	1	1270.G42	1	1	0
*Sinuber* sp. 1	1	JR15005_91	1	1893.G38	1	1	0
*Marseniopsis* sp. 1	1	JR15005_91	1	1893.G39	1	1	0
*Marseniopsis* sp. 2	1	JR15005_91	1	1894.G43	1	1	0
*Marseniopsis* sp. 3	1	JR15005_68	1	1306.G34	1	1	0
*Antarctodomusthielei*	1	JR15005_101	1	2117.G17	1	1	0
*Chlanidotasigneyana*	27	JR15005_17	1	0337.G03	1	1	0
		JR15005_32	1	0647.G03	1	1	0
		JR15005_44	3	0892.G03	3	2	1
		JR15005_45	1	0931.G03	1	1	0
		JR15005_61	8	1106.G03	1	1	0
				1133.G03	7	3	4
		JR15005_62	1	1163.G03	1	1	0
		JR15005_63	8	1264.G03	2	1	1
				1270.G03	6	4	2
		JR15005_68	1	1306.G03	1	1	0
		JR15005_70	1	1486.G03	1	0	1
		JR15005_83	1	1747.G03	1	1	0
		JR15005_132	1	2841.G03	1	1	0
*Chlanificulathielei*	15	JR15005_15	1	0273.G04	1	0	1
		JR15005_17	2	0337.G04	2	0	2
		JR15005_63	1	1270.G04	1	0	1
		JR15005_68	1	1339.G04	1	0	1
		JR15005_83	1	1747.G04	1	1	0
		JR15005_85	2	1792.G04	2	1	1
		JR15005_91	3	1893.G04	3	0	3
		JR15005_93	1	1985.G04	1	0	1
		JR15005_94	2	2010.G04	1	0	1
				2011.G04	1	1	0
		JR15005_130	1	2788.G04	1	0	1
*Neobuccinumeatoni*	4	JR15005_31	1	0551.G19	1	1	0
		JR15005_61	2	1133.G19	2	1	1
		JR15005_63	1	1270.G19	1	1	0
*Probuccinumtenerum*	1	JR15005_119	1	2525.G13	1	1	0
*Falsitromina* sp. 1	2	JR15005_63	2	1264.G29	2	2	0
*Trophoncoulmanensis*	4	JR15005_61	4	1133.G23	4	4	0
*Trophon* sp. 1	1	JR15005_44	1	0892.G33	1	1	0
*Volvarinaealesae*	3	JR15005_44	1	0892.G12	1	1	0
		JR15005_93	1	1960.G12	1	1	0
		JR15005_119	1	2525.G12	1	1	0
*Volvarinahyalina*	1	JR15005_44	1	0892.G20	1	1	0
*Harpovolutacharcoti*	33	JR15005_55	3	1050.G02	1	1	0
				1051.G02	1	1	0
				1052.G02	1	1	0
		JR15005_61	4	1074.G02	1	1	0
				1133.G02	3	3	0
		JR15005_62	7	1148.G02	1	1	0
				1149.G02	1	1	0
				1150.G02	1	1	0
				1163.G02	4	1	3
		JR15005_63	7	1251.G02	1	1	0
				1252.G02	1	1	0
				1253.G02	1	1	0
				1254.G02	1	1	0
				1255.G02	1	1	0
				1264.G02	1	1	0
				1281.G02	1	1	0
		JR15005_69	1	1420.G02	1	1	0
		JR15005_79	1	1598.G02	1	1	0
		JR15005_93	3	1955.G02	1	1	0
				1957.G02	1	1	0
				1959.G02	1	1	0
		JR15005_94	1	1996.G02	1	1	0
		JR15005_95	3	2067.G02	1	1	0
				2068.G02	1	1	0
				2069.G02	1	1	0
		JR15005_126	2	2712.G02	1	1	0
				2713.G02	1	1	0
		JR15005_132	1	2855.G02	1	1	0
*Miomelonturnerae*	4	JR15005_15	1	0273.G08	1	0	1
		JR15005_83	2	1747.G08	2	1	1
		JR15005_85	1	1792.G08	1	1	0
*Tractolira* sp. 1	1	JR15005_63	1	1264.G26	1	1	0
Paradmetecf.fragillima	6	JR15005_43	1	0843.G22	1	1	0
		JR15005_44	5	0892.G22	5	3	2
*Paradmetepercarinata*	10	JR15005_16	1	0296.G10	1	1	0
		JR15005_61	4	1133.G10	4	3	1
		JR15005_62	1	1163.G10	1	1	0
		JR15005_63	2	1264.G10	1	0	1
				1270.G10	1	0	1
		JR15005_83	1	1747.G10	1	1	0
		JR15005_85	1	1792.G10	1	1	0
*Paradmete* sp. 1	1	JR15005_44	1	0892.G21	1	1	0
Belaturriculacf.ergata	1	JR15005_132	1	2841.G31	1	1	0
*Belaturriculagaini*	3	JR15005_44	1	0892.G18	1	0	1
		JR15005_112	1	2290.G18	1	1	0
		JR15005_126	1	2750.G18	1	1	0
*Typhlodaphneparatenoceras*	10	JR15005_15	1	0273.G09	1	1	0
		JR15005_61	1	1133.G09	1	0	1
		JR15005_62	2	1163.G09	2	1	1
		JR15005_79	1	1600.G09	1	0	1
		JR15005_83	1	1747.G09	1	1	0
		JR15005_85	2	1792.G09	2	2	0
		JR15005_112	1	2290.G09	1	0	1
		JR15005_113	1	2387.G09	1	0	1
Neogastropoda cf. Borsoniidae sp. 1	2	JR15005_112	1	2290.G37	1	1	0
		JR15005_119	1	2533.G37	1	1	0
*Lorabelapelseneeri*	1	JR15005_6	1	0152.G14	1	1	0
Pleurotomellacf.simillima	1	JR15005_63	1	1264.G28	1	1	0
*Aforiamagnifica*	15	JR15005_15	1	0273.G06	1	0	1
		JR15005_33	1	0728.G06	1	1	0
		JR15005_61	4	1133.G06	4	2	2
		JR15005_62	2	1163.G06	2	2	0
		JR15005_70	1	1486.G06	1	0	1
		JR15005_79	2	1600.G06	2	0	2
		JR15005_90	1	1832.G06	1	1	0
		JR15005_91	2	1893.G06	2	2	0
		JR15005_113	1	2387.G06	1	1	0
*Conorbelaantarctica*	23	JR15005_15	1	0273.G05	1	1	0
		JR15005_61	9	1133.G05	9	5	4
		JR15005_62	3	1163.G05	3	2	1
		JR15005_63	6	1264.G05	2	1	1
				1270.G05	4	2	2
		JR15005_70	1	1486.G05	1	0	1
		JR15005_85	3	1792.G05	3	3	0
*Micropleurotoma* sp. 1	9	JR15005_61	5	1133.G15	5	1	4
		JR15005_63	4	1264.G15	3	2	1
				1270.G15	1	0	1
*Nothoadmeteconsobrina*	3	JR15005_61	1	1133.G27	1	1	0
		JR15005_63	2	1264.G27	2	2	0
*Neactaeoninaedentula*	3	JR15005_54	1	1029.G24	1	0	1
		JR15005_61	1	1133.G24	1	1	0
		JR15005_100	1	2102.G24	1	1	0
*Newnesia* sp. 1	3	JR15005_105	1	2225.G44	1	1	0
		JR15005_138	2	2903.G44	2	2	0
*Philine* sp. 1	4	JR15005_15	1	0363.G11	1	1	0
		JR15005_16	1	0312.G11	1	1	0
		JR15005_17	2	0335.G11	2	2	0
Prodoriscf.clavigera	2	JR15005_113	1	2386.G66	1	1	0
		JR15005_125	1	2672.G66	1	1	0
*Doriskerguelenensis*	5	JR15005_33	2	0709.G62	2	2	0
		JR15005_91	1	1896.G62	1	1	0
		JR15005_113	1	2386.G62	1	1	0
		JR15005_114	1	2457.G62	1	1	0
Nudibranchia sp. 1	2	JR15005_79	1	1613.G63	1	1	0
		JR15005_81	1	1694.G63	1	1	0
Nudibranchia sp. 2	1	JR15005_81	1	1694.G64	1	1	0
Nudibranchia sp. 3	1	JR15005_94	1	1998.G65	1	1	0
*Dentaliummajorinum*	15	JR15005_61	2	1132.S01	2	1	1
		JR15005_63	3	1263.S01	3	2	1
		JR15005_68	1	1343.S01	1	0	1
		JR15005_70	6	1469.S01	6	5	1
		JR15005_79	1	1600.S01	1	0	1
		JR15005_91	2	1895.S01	2	2	0
*Siphonodentaliumdalli*	105	JR15005_6	77	0155.S02	77	75	2
		JR15005_61	6	1132.S02	6	6	0
		JR15005_62	1	1162.S02	1	1	0
		JR15005_63	21	1263.S02	21	21	0
